# Efficacy and safety of blood purification in the treatment of deep burns: A systematic review and meta-analysis

**DOI:** 10.1097/MD.0000000000023968

**Published:** 2021-02-05

**Authors:** Gaofei Zhang, Wenjun Liu, Jiamei Li, Di Wang, Jianxing Duan, Hanxiao Luo

**Affiliations:** a Department of Burn and Injury; b Department of Burn and Injury, Second Affiliated Hospital of Kunming Medical University, Kunming City, Yunnan, China.

**Keywords:** blood purification, deep burn, hemofiltration, meta-analysis, plasmapheresis, sepsis

## Abstract

**Introduction:**

This meta-analysis aimed to systematically review and evaluate randomized controlled trials (RCTs) and cohort studies examining the efficacy and safety of blood purification in the treatment of patients with deep burns.

**Methods:**

The PubMed, Cochrane Library, and Embase databases and relevant references were systematically searched for RCTs and cohort studies published until the end of September 2020 to investigate the potential of blood purification in improving the prognosis of severely burned patients. The primary outcome of this systematic review was overall patient mortality; secondary outcomes included the incidence of sepsis and infection prevention (vital signs and routine blood tests).

**Results:**

A total of 6 RCTs and 1 cohort study were included, with a total of 538 burn patients (274 patients who received blood purification and 264 control patients). Compared with patients who received conventional treatment, those treated with blood purification displayed significant 2-day reduction in mortality and sepsis with relative risks of 0.62 and 0.41, respectively (95% confidence intervals [CIs], 0.74–0.82 and 0.25–0.67, respectively; *P* < .05). In terms of vital signs and blood biochemistry, the respiratory rates and blood urea nitrogen levels of patients in the blood purification group 3 days post-treatment were significantly higher than those in the control group (randomized standard deviations (SMDs), 0.78 and 0.77, respectively; 95% CIs, 0.33–1.23 and 1.22–0.31, respectively; *P* < .05). However, there were no significant differences between groups on day 3 with regard to temperature (*P* = .32), heart rate (*P* = .26), white blood cell count (*P* = .54), or neutrophil count (*P* = .74), potentially owing to the small sample size or the relatively short intervention time. Heterogeneous differences existed between the groups with respect to blood urea nitrogen (SMD = -1.22; 95% CI, -2.16 to -0.40; *P* < .00001) and Cr (SMD = -3.13; 95% CI, -4.92 to -1.33; *P* < .00001) on day 7. No systematic adverse events occurred.

**Conclusions:**

Blood purification treatment for deep burn patients can significantly reduce the mortality rate and the incidence of complications.

## 1 Introduction

Severe burns can occur at any time in day-to-day life. Their chances of occurrence increase with the development of industrial and economic systems owing to natural or man-made accidents in work and home environments.^[1]^ An effective method of treating burns is desperately needed to reduce the incidences of related mortality and complications. There is considerable evidence that sepsis in severely burned patients is associated with an inflammatory state. Thus, a non-selective approach via extracorporeal blood purification is an attractive treatment option until the pathophysiology of the inflammatory response related to burns is more fully understood.^[2]^ It has been reported that early intervention with blood purification reduces the incidence of sepsis, septic shock, and organ failure in patients with burns ≥ 50% total body surface area and improves the survival of patients with burns ≥ 80% total body surface area.^[3]^ Blood purification includes hemofiltration and plasmapheresis.

Blood purification treatment can also nonspecifically remove inflammatory mediators in blood circulation through filtration, adsorption, plasmapheresis, and other means; this can help maintain the balance of pro-inflammatory and anti-inflammatory reactions.^[4]^ Therefore, blood purification therapy is suitable for patients with excessive systemic inflammation and is beneficial for the treatment of burn sepsis.^[5]^ The primary methods of early blood purification are filtration adsorption through continuous renal replacement therapy, which is used to clear renal failure in vivo metabolite and moisture. With the development of blood purification technology, the current mode of continuous veno-venous hemofiltration (CVVH/CVVHDF) is used to remove inflammatory mediators, cytokines, chemokines, bradykinin, and leukotrienes and can help reduce cytotoxicity by non-specific adsorption. High-volume hemofiltration is based on the same principle as that of high-dose CVVH. By increasing the volume of replacement fluid, High-volume hemofiltration enhances convection and adsorption of small- and medium-molecular solutes and improves solute clearance. Plasmapheresis is a treatment option characterized by clarification of metabolites in the body; it also maintains the balance of water and electrolytes. During plasmapheresis, blood cells and plasma are separated so that the patient's plasma can be treated separately. It is a blood purification method that can remove macromolecular substances present in blood, and it can be combined with hemofiltration to enhance the removal of small- and medium-weight therapeutic factors. Although hemofiltration and plasmapheresis are separate methods of blood purification, their purposes are the same—to avoid an excessive inflammatory response and maintain the anti-infection ability of the body during infection periods by removing inflammatory mediators, oxygen free radicals, and other pathogenic mediators that are present in the blood.^[6]^ Therefore, plasmapheresis and hemofiltration can be studied together. In summary, attempts to moderate inflammatory reactions, regulate cytokine homeostasis, and decrease myoglobin level by blood purification in the early stages of burn trauma seem to be promising therapeutic options.^[2]^

However, blood purification as a specialized technique requires specific equipment and extra training. Theoretically, it could have harmful effects on a patient's blood pressure or could remove beneficial substances (such as antibiotics) from the blood.^[7]^ Consequently, we conducted a systematic review of the literature to assess whether blood purification is associated with reduced mortality and sepsis rates among patients with severe burns. We also aimed to assess whether blood purification can improve vital signs and routine blood laboratory values in patients with severe burns.

## 2 Methods

### 2.1 Search strategy

This meta-analysis conformed to the Preferred Reporting Items for Systematic Reviews and Meta-Analyses Statement. We included randomized controlled trials (RCTs) and cohort studies from the searchable online PubMed, Embase, and Cochrane Library databases, accessible on or before September 10, 2020. We searched publications on the effects of blood purification compared to those of traditional treatments for deep burns. The search criteria were as follows:

1) “burn” and
2) “hemofiltration” OR “blood purification” OR “plasmapheresis.”

PubMed medical subject headings (MeSH) standards were also used for the following searches:

(1) “Burns”[Mesh] AND “Hemofiltration”[Mesh],
(2) “Burns”[Mesh] AND “Plasma Exchange”[Mesh], and
(3) “Plasmapheresis”[Mesh] AND “Burns”[Mesh].

This was a clinical study of only human subjects and included an intervention group (those who underwent blood purification) and a control group (those who received conventional treatment). We also analyzed references to articles that did not meet the inclusion criteria as well as references to review articles and included any references that met the inclusion criteria in the study. As this study was a meta-analysis, it involved only the analysis of data in the network database; therefore, no ethical approval was required.

### 2.2 Inclusion and exclusion criteria

The studies were considered for inclusion if they met all of the following criteria:

(1) RCTs and cohort studies comparing plasmapheresis or hemofiltration with conventional treatments;
(2) the experimental group comprised patients who received “hemofiltration (whether continuous or discontinuous), plasmapheresis, or blood purification,” and participants in the control group received “standard or routine” treatment and did not receive similar hemofiltration;
(3) RCTs and cohort studies without restrictions on patients’ race, sex, and disease or cause of injury; and
(4) RCTs reporting at least 1 of the outcomes of interests including mortality, incidence of sepsis, or vital signs (respiratory rate, body temperature and heart rate), and routine blood test results (White blood cell [WBC] counts, blood urea nitrogen [BUN] and creatinine [Cr] levels).

The following types of studies were excluded: republished articles, meeting minutes or abstracts; we also excluded studies that had patients who were younger than 16 years old and studies that did not include patients with deep burns.

### 2.3 Data extraction and management

Two review authors (GZ and WL) independently extracted the data using the revised Cochrane data extraction format. The first reviewer performed the calculation, and the second reviewer reviewed the calculation. Differences were resolved through discussion between the authors. The names of study authors, researchers, or institutions and the results of the study were not blinded. We listed exclusion trials and reasons for exclusion. We experimented with the data extraction form before using it. The extracted data included the first author name, publication year, study design, total sample size, blood purification treatment type, deep burn type, control group treatment type, outcome, and adverse reactions from each study. Outcomes were mortality rates, sepsis rates, vital signs (respiratory rate, body temperature and heart rate) and routine blood test results (WBC counts, BUN and Gr levels).

### 2.4 Quality assessment

Studies that met the inclusion criteria were assessed for risk of bias according to the Cochrane Collaboration methods. Quality evaluation was carried out independently by 2 researchers and cross-checked. In case of differences, a third researcher was invited to settle disagreements. Finally, a summary chart of risk of bias was made.

### 2.5 Statistical analysis

The standardized mean differences (SMDs) were calculated for continuous variables, and the relative risks (RRs) were used as the effect sizes for the classification variables, with α = 0.05 as the test standard. For individual studies with specific interventions and statistical indicators, only the effect sizes and 95% CIs were calculated, and then descriptive analysis was performed to assess the differences for hemofiltration in mortality, sepsis, routine blood test results, and vital signs in patients with deep burns. Statistical heterogeneity was analyzed using I^2^ statistics. The fixed-effects model was used when *I*^2^ < 50%, and the random-effects model was used when *I*^*2*^ ≥50%. If there was significant heterogeneity in the cause of injury, sensitivity analysis or subgroup analysis was performed. The effects of publication bias and small-scale studies were evaluated by Egger and Begg tests. Analyses were performed using Stata 15.1 software (Stata Corporation) and Review Manager 5.3 software (the Cochrane Collaboration).

## 3 Results

### 3.1 Description of the included studies

**Figure 1:**
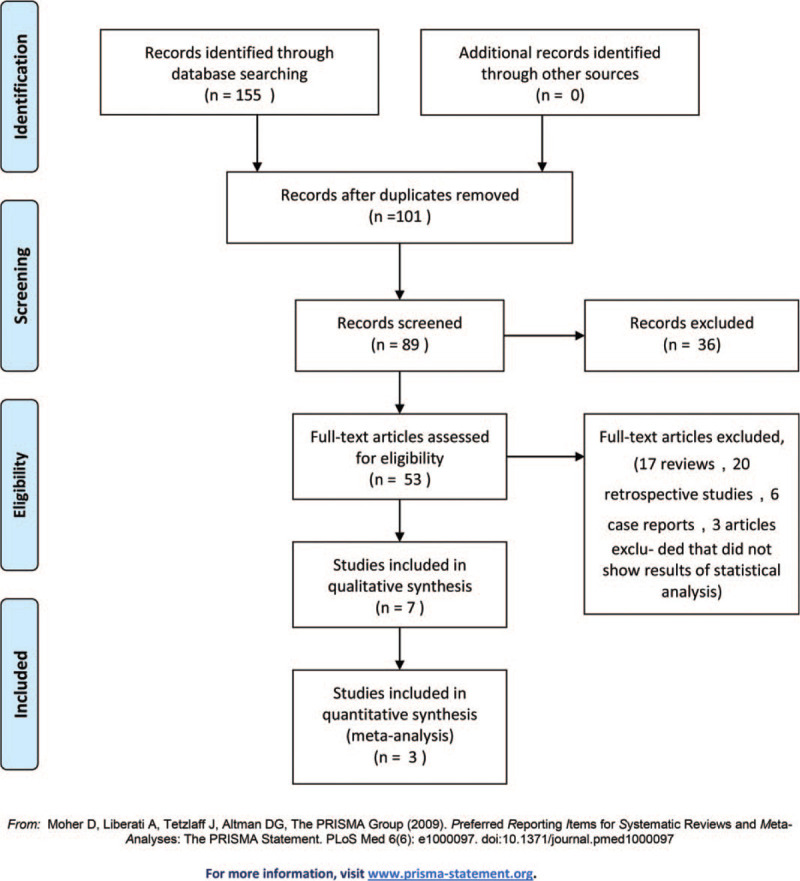
Flow diagram of the study selection process.

A total of 235 studies were initially retrieved from PubMed (n = 113), Cochrane Library (n = 12), and Embase (n = 110), which were screened thoroughly. Ultimately, 7 studies (6 RCTs and 1 cohort study) were included. Information regarding the included studies is shown in Fig. 1. These studies included a total of 538 burn patients (274 patients who underwent blood purification and 264 control patients) (Table 1).

**Table 1:** Details and outcome measures of the seven studies included in the meta-analysis.

First author, year and group	Number of patients	Sex (M/F)	Mean age (yr)	Number of wounds	TBSA (%)	Intervention	Outcome measures of the studies
Lyu, T, 2018							
Experimental	43	29/14	34 ± 4	43	20 ± 3	routine treatment+ The next day, continuous plasma filtration adsorption (discontinued at 7 d)	①③⑤
Control	43	30/13	35 ± 5	43	20 ± 3	routine treatment (discontinued at 7 d)	①③⑤
You, B, 2018							
Experimental	41	31/10	39.6 ± 10.6	41	55-85	routine treatment+ Early excision and skin graft + HVHF therapy (a 1.5-m2 haemofilter , Blood flow was set between 200 and 250 mL/min , the ultrafiltration flow was 65 mL/kg/h., discontinued at 3 d)	①②③
Control	41	34/7	42.3 ± 12.0	41	60-82	routine treatment+ Early excision and skin graft	①②③
Chung, K.2017							
Experimental	23	17/6	42-60	23	30-60	routine treatment + RRT + CVVH (70 ml/kg/h)	①③
Control	14	11/3	37-62	14	29-58	routine treatment + RRT (20–35 ml/kg/h)	①③
Liu, F, 2016							
Experimental	20	16/4	41 ± 11	20	74 ± 16	routine treatment (CT)+venous-venous hemodiafiltration (Blood flow was set between 150 and 200 ml/min,the ultrafiltration flow was 55–60 mL/kg/h. Within 72 hours after sustained injury)	①②③④⑤
Control	21	20/1	46 ± 9	21	72 ± 14	routine treatment (CT)	①②③④⑤
Zu, H, 2015							
Experimental	98	72/26	46.2 ± 8.7	98		routine treatment+ CVVHDF (Blood flow was set between 200 and 250 ml/min,liquid volume of 2.5–3.0 L/h. Blood purification lasting over 8–10 h)	①③
Control	97	70/27	47.1 ± 9.4	97		routine treatment	①③
Guo, W, 2015							
Experimental	20	13/7	31 ± 7	20	78 ± 20	routine treatment (CT)+Early excision and skin graft +intermittent hemofiltration combined with HP (Blood flow was set between 150 and 300 mL/min)	①②④⑤
Control	20	14/6	31 ± 7	20	76 ± 18	routine treatment (CT) + Early excision and skin graft	①②④⑤
Chung, K,2009							
Experimental	29		27 ± 8	29	64 ± 18	routine treatment +CVVH (the ultrafiltration flow was 57 ± 19 ml/kg/h.)	①③
Control	28		38 ± 18	28	58 ± 18	routine treatment	①③

### 3.2 Risk of bias assessment

**Figure 2:**
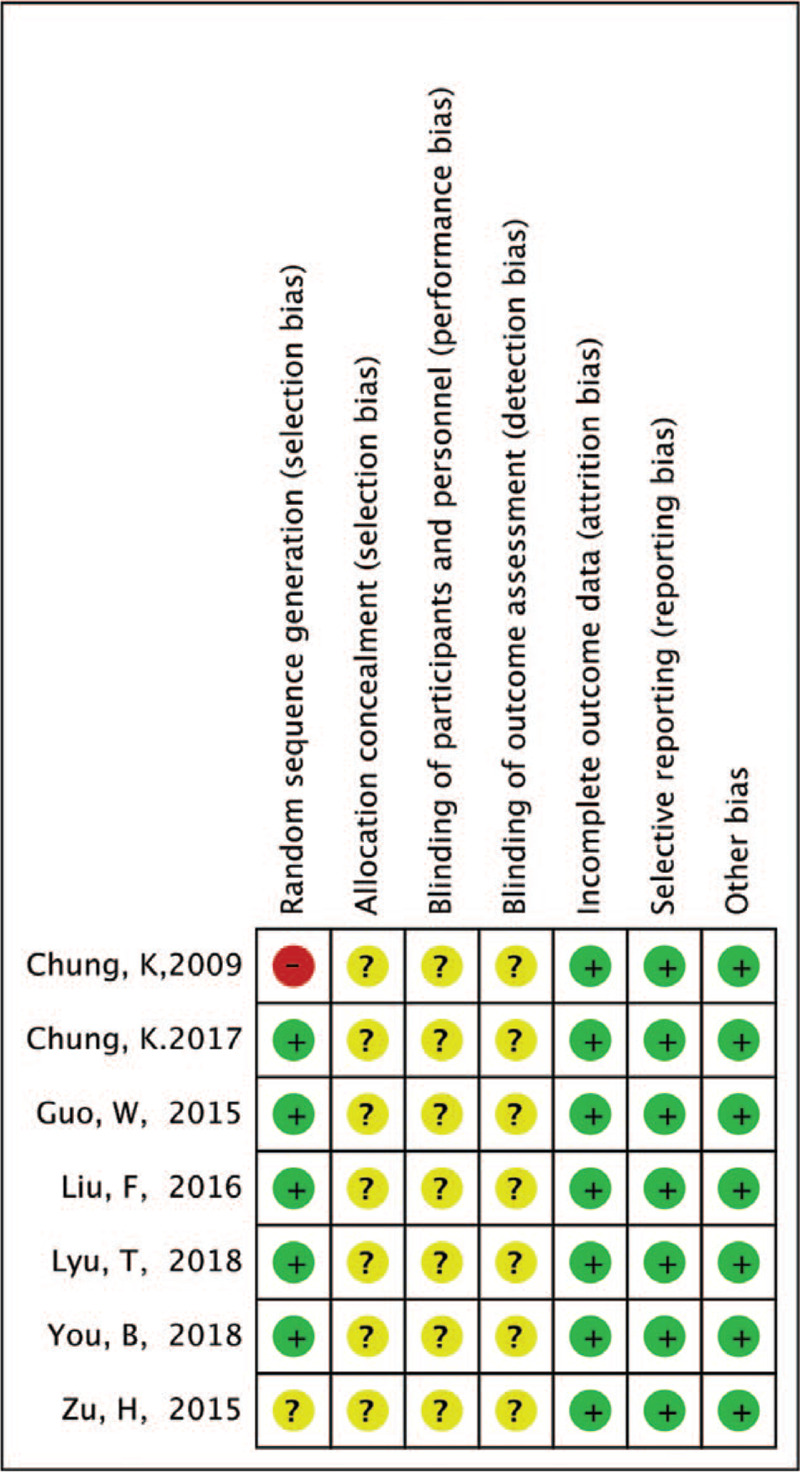
Risk of bias of the included 7 studies.

Among the 7 included studies, 6 were RCTs,^[3,7–11]^ and 1 was a cohort study.^[12]^ Five trials explained the methods of random sequence generation. The allocation concealment of all included studies was uncertain (Fig. 2). Blinding of the outcome assessment was uncertain for all the included studies. All studies had a low risk of bias with regard to incomplete outcome data and selective reporting.

### 3.3 Outcomes

#### 3.3.1 Mortality at day 28

**Figure 3:**
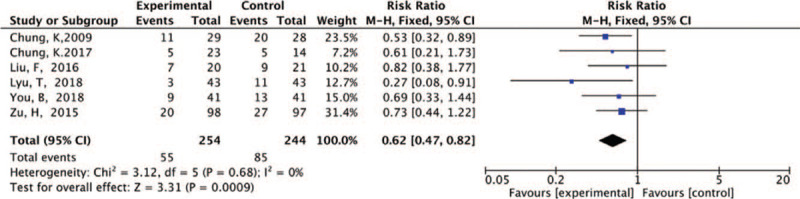
Meta-analysis of 28-day mortality in both groups.

The meta-analysis of the mortality at day 28 included 498 patients with burns (254 patients who underwent blood purification and 244 control patients) from 6 studies. The fixed-effects analysis (*I*^2^ = 0%; *P* = .68) revealed that the mortality from the beginning of the trial to day 28 was significantly lower in the blood purification group than in the control group (RR = 0.62; 95% CI, 0.74 to 0.82; *P* = .0009) (Fig. 3).

#### 3.3.2 Incidence of sepsis

**Figure 4:**
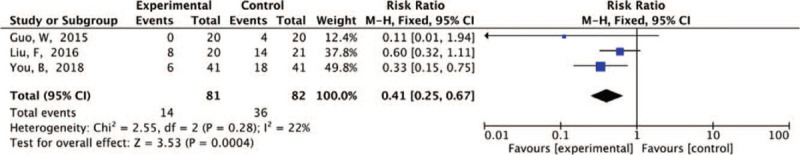
Meta-analysis of the incidence of sepsis in both groups.

The number of patients reported to have sepsis in 3 studies was 163 (81 in the blood purification group and 82 in the control group). There was no significant heterogeneity among the results (*I*^2^ = 22%; *P* = .28). The results of the fixed-effects model meta-analysis showed that the incidence of sepsis in deep burn patients treated with blood purification was lower than that in patients treated with conventional therapy (RR = 0.41; 95% CI, 0.25–0.67; *P* = .0004) (Fig. 4).

#### 3.3.3 Safety (vital signs and routine blood laboratory results)

Three studies concluded that blood purification treatment for severe burns had no adverse effect on patient vital signs and was safe and feasible ^[7,9,11]^. No systemic adverse events were observed.

#### 3.3.4 Respiratory rate at 3 days

**Figure 5:**
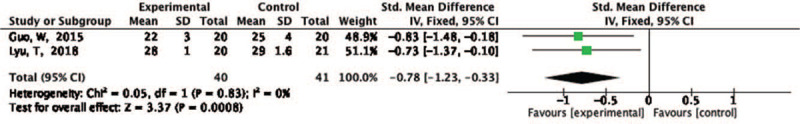
Meta-analysis of respiratory rates in both groups.

A meta-analysis of respiratory rates on day 3 included 81 burn patients from 2 RCTs (40 patients treated with blood purification and 41 control patients). Analysis of fixed effects showed that from the beginning of the trial to day 3, the respiratory rates of patients in the blood purification group were significantly lower than rates for patients in the control group (SMD = -0.78; 95% CI, -1.23 to -0.33; *P* = .0008) (Fig. 5).

#### 3.3.5 BUN levels at 3 days

**Figure 6:**
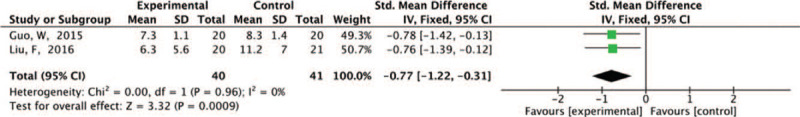
Meta-analysis of BUN in both groups at 3 days (abbreviations: BUN, blood urea nitrogen).

The meta-analysis of BUN levels on day 3 included 81 patients with burns (40 patients who received blood purification and 41 control patients) from 2 RCTs. The fixed-effects analysis (*I*^2^ = 0%, *P* = .96) showed that BUN levels from the beginning of the trial to day 3 were significantly lower in the blood purification group than in the control group (SMD = 0.77; 95% CI, -1.22 to -0.31; *P* = .0009) (Fig. 6).

#### 3.3.6 WBC counts at 7 days

**Figure 7:**
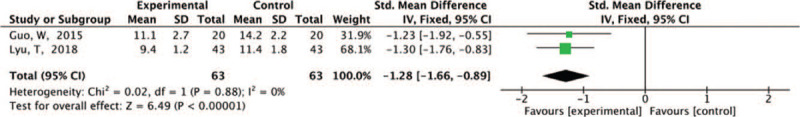
Meta-analysis of WBC in both groups at 7 days (abbreviations: WBC, white blood cells).

A meta-analysis of WBC counts on day 7 included 81 burn patients from 2 RCTS (40 patients treated with blood purification and 41 control patients). There was no significant heterogeneity among results (*I*^2^ = 0%; *P* = .88). The results of the fixed-effects model meta-analysis showed that the WBC counts at 7 days in patients treated with blood purification were lower than those in the control group patients at 3 days of treatment (SMD = -1.28; 95% CI, -1.66 to -0.89; *P* < .001) (Fig. 7).

### 3.4 Assessment of bias

**Figure 8:**
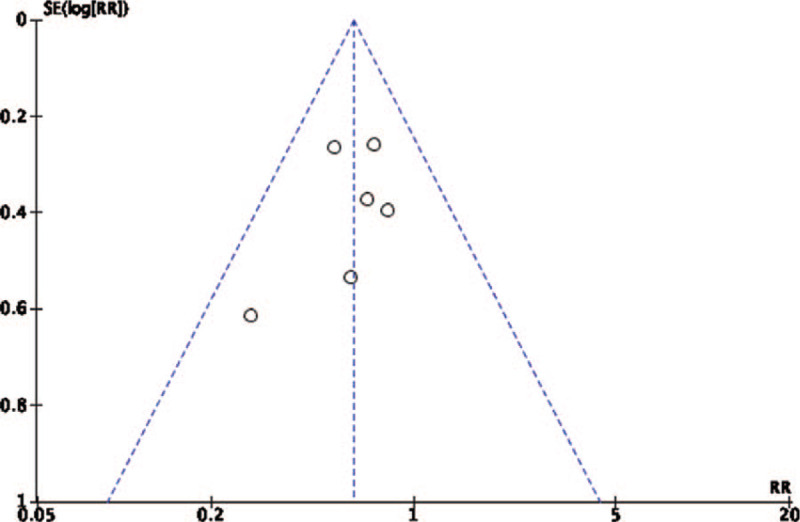
Funnel plot (effect size on the Y-axis and standard error of effect size on the X-axis) showing no significant publication bias.

Egger and Begg tests indicated possible publication bias and small-study effects for respiratory rate at 3 days, BUN levels at 3 days after treatment, and WBC counts at 7 days after treatment (*P* < .05 and *P* = 1.000 for all). No significant publication bias regarding mortality was observed on day 28 when assessed using Begg test and the trim and fill method. (Fig. 8)

## 4 Discussion

Severe burns and sepsis are associated with poor patient outcomes. Blood purification treatment is thought to confer potential benefits for these patient. This systematic review showed that despite the potential benefits of blood purification, very few studies have been performed to investigate its use in patients with severe burns and sepsis. In the recent meta-analysis by Zhang et al, hemofiltration was used to evaluate sepsis patients; however, they concluded that the data were insufficient to assess the prognosis of patients^[13]^ Their study was conducted among intensive care unit patients, rather than deep burn patients specifically.

**Figure 9:**
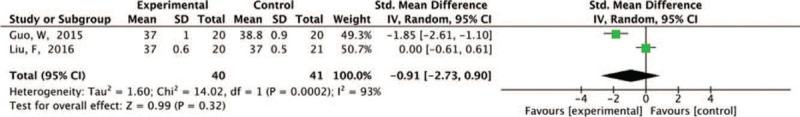
Meta-analysis of body temperature in both groups at 3 days.

**Figure 10:**
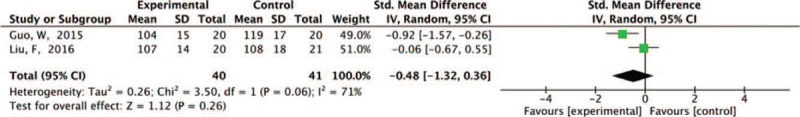
Meta-analysis of heart rate in both groups at 3 days.

**Figure 11:**
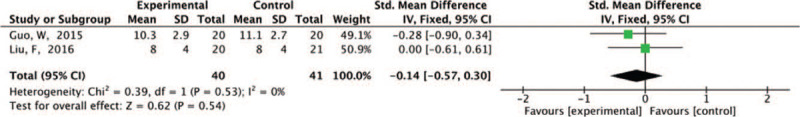
Meta-analysis of WBC counts in both groups at 3 day (abbreviation WBC, white blood cell).

**Figure 12:**
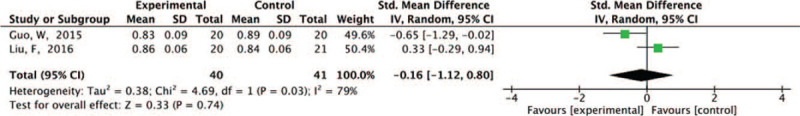
Meta-analysis of NE in both groups at 3 day (abbreviation: NE, neutrophils).

The present meta-analysis showed that in comparison with traditional treatment:

(1) The mortality rate at day 28 and incidence of sepsis were significantly reduced in the blood purification group compared with rates observed for the control group;
(2) at the beginning of the treatment, from days 3 to 7, the vital signs and blood biochemical indicators of patients in the blood purification group were lower than those of patients in the control group, indicating that blood purification treatment for deep burn patients may affect vital signs and blood biochemical indicators.
(3) However, there were no significant differences in body temperature, heart rate, WBC counts, or neutrophil counts between the control patients and the blood purification patients on day 3 after blood purification intervention (Figs. 9–12).

**Figure 13:**
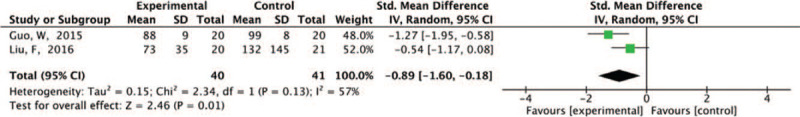
Meta-analysis of Cr in both groups at 3 days.

**Figure 14:**
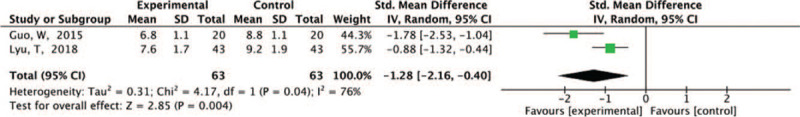
Meta-analysis of BUN in both groups at 7 days (abbreviation: BUN, blood urea nitrogen).

**Figure 15:**
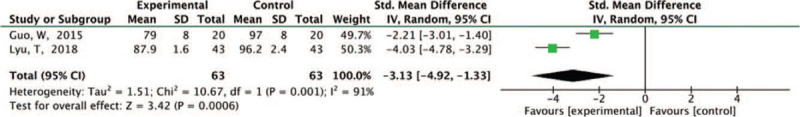
Meta-analysis of Cr in both groups at 7 days.

Additionally, in the blood purification group, the statistical results regarding Cr on day 3 after blood purification intervention showed greater heterogeneity when compared with those of the control group (Fig. 13). Furthermore, the statistical results regarding BUN and Cr for the blood purification group were heterogeneous compared with those for the control group on day 7 after blood purification intervention (Figs. 14 and 15). The reasons for this finding could be as follows: blood purification does not remove certain heat-causing factors or neurotransmitters; thus, it does not affect body temperature, heart rate, and other vital signs, which require further verification. On the basis of the current RCTs, a conclusion cannot be drawn as to whether blood purification treatment in the early stages of severe burn treatment will have a benign effect on the vital signs, fluid rehydration, and urine quantity of patients. However, according to studies published by Guo et al^[12]^ and Liu et al,^[7,9]^ the early pro-inflammatory cytokines in the hemofiltration group consisted of IL-1, IL-6, and tumor necrosis factor, which were significantly lower than those in the control group, further indicating that blood purification treatment can reduce the inflammatory responses of burn patients. Therefore, this current systematic review highlights the continued lack of evidence in this field and the need for additional large-scale clinical trials to determine the effects of blood purification on prognosis.

The limitations of this study are as follows. First, the risk of RCT bias is high because RCTs are not specific to blind and random sequence generation methods. Second, studies with results that were inconsistent with those found in this study were not available in the literature; therefore, this may have led to bias in our analysis. Third, bias may exist due to the large publication time span of the included studies. Fourth, although the number of cases was sufficient, the number of RCTs was small, which possibly led to bias.

## 5 Conclusions

Our study found that early blood purification treatment for patients with severe burns reduced the rates of mortality and sepsis and improved overall outcomes. We have not found any evidence that such therapeutic interventions are harmful. Therefore, blood purification interventions should be actively used early in the treatment process for patients with severe burns (rather than implementing passive organ support treatments when organ failure occurs). However, this study also showed that the effects of blood purification treatment were not as significant as expected, and blood purification did not completely change the outcome of severe complications. Few studies have investigated the use of blood purification in patients with severe burns (7 studies, 538 participants, low-quality evidence). Researchers should consider more RCTs, that include large, multicenter trials to produce more clinically relevant results.

## Author contributions

**Data curation:** Jianxing Duan.

**Investigation:** Jiamei Li, Jianxing Duan.

**Methodology:** Gaofei Zhang, Wenjun Liu, Di Wang, Hanxiao Lou.

**Validation:** Hanxiao Luo.

**Writing – original draft:** Gaofei Zhang.

**Writing – review & editing:** Gaofei Zhang.
